# Contraceptive Use among Basic School Pupils in Ghana: A Case Study of a Municipality

**DOI:** 10.1155/2020/7521096

**Published:** 2020-09-23

**Authors:** Fred Yao Gbagbo

**Affiliations:** Department of Health Administration and Education, Faculty of Science Education, University of Education, Winneba, P.O. Box 25, Winneba, Central Region, Ghana

## Abstract

**Background:**

Ghana over the years strived to improve contraceptive services for young people through various policies and programs. Despite these efforts, contraceptive use among young people remains a challenge. In this study, contraceptive use among basic school pupils in a Ghanaian municipality was explored to inform policy and program decisions.

**Methods:**

The research design was a cross-sectional and mixed-method survey involving four hundred and twenty-seven (427) respondents randomly selected from four hundred and eleven (411) basic schools (102 from private and 309 from public basic schools) within Effutu Municipality of Ghana.

**Results:**

Basic school pupils in Ghana are generally sexually active but have high unmet needs for modern contraception due to sociocultural barriers, stigma, and misconceptions. Awareness and use are however more prevalent among junior high school pupils compared to those at the primary levels. Pupils who received contraceptive education from parents/guardians were, however, more likely to use modern contraceptives consistently than their counterparts who do not.

**Conclusions:**

Because young people in basic schools are becoming sexually active, there is a need for formalized contraceptive education in basic schools for correct information and education.

## 1. Background

Reproductive health issues have been a global concern since the past decade [1], yet efforts to achieve the millennium development goals (MDGs) 5 have only made adolescent reproductive health issues only more prominent [2], without achieving remarkable global results. Consequently, the succeeding sustainable development goal (SDG) 3 has emphasized the crucial role of adolescent reproductive health research towards achieving the tenants of the sustainable development goal (SDG) 3 since reproductive health (RH) affects the total well-being of young people as they grow into adulthood [3]. Empirical evidence in recent times has shown that more than half of the world population is under the age of twenty-five years with one in four under age eighteen and in basic schools [4].

The basic school concept in Ghana comprises primary school and junior high school (JHS) for children from about four to eleven years old, in which they receive primary or elementary education. Usually, it comes after preschool and before secondary school [5]. In Ghana, adolescent reproductive health is a great concern to the nation [6, 7]. This concern has been examined by researchers that observed that early commencement of sexual activities, poor knowledge of contraceptives, and limited access to and underutilization of reproductive health services are key challenges confronting national adolescent reproductive health initiatives [8] as poor knowledge of contraceptives among adolescents have been linked incomplete information and poor access resulting to a high prevalence of sexually transmitted infections (STIs), unwanted pregnancies, and other maternal health problems among young people [9].

To ensure that young people are well educated to make informed decisions on their sexuality, the Nana Akufo Addo administration of the Republic of Ghana, in 2019, proposed a comprehensive sexuality education initiative in basic schools. The researches in this study, therefore, seek to explore awareness, knowledge, and contraceptive use of basic school pupils to inform policy and program decisions on contraception among the young people in Ghana.

## 2. Methods

### 2.1. Study Design

The research design was a cross-sectional survey using a mixed-method (qualitative and quantitative) approach for data collection. The qualitative component focused on the experiences of respondents in relation to the research topic whilst the quantitative component examined the background characteristics of the respondents involved.

### 2.2. Setting and Population

The study was conducted in the Effutu Municipality of the central region of Ghana. It is one of the twenty [10] districts in the Central Region of Ghana with its capital as Winneba. The population of the Effutu Municipality, according to the 2017 Annual Report of the Municipal Health Directorate, is 79,411. The study population comprises basic school pupils in Effutu Municipality. According to the Effutu Municipal Education Service, there were 49 basic schools (27private and 22 public) in the study area as of December 2019 with a total basic school pupil's population of two hundred and fifty-three thousand (253,000). These constitute the sample frame from which the pupils were sampled for the study.

## 3. Inclusion Criteria

Participants were strictly from basic schools (public or private) known by the Municipal Education Service and have primary and junior high school pupils aged 10 to 18+ years.

### 3.1. Sampling

Four hundred and eleven (411) basic school pupils (102 from private and 309 from public basic schools) in upper primary to JHS 3 within Effutu Municipality were randomly selected and interviewed using a structured questionnaire. This sample size was estimated using an online Raosoft sample size calculator [11]. The sample size estimation was based on the available total population of basic school pupils in the 49 identified schools in the metropolis (253,000) as of October 2019, using 95% confidence interval, 5% margin of error, and 50% response distribution. In terms of the figures, the sample size *n* and margin of error *E* are given by

(1) $$\begin{array}{c} \begin{array}{c} x=Z{\left(\frac{c}{100}\right)}^{2}r\left(100-r\right)\\ n=\frac{Nx}{\left(\left(N-1\right)E^{2}+x\right)}\\ E=Sqrt\left[\frac{\left(N-n\right)x}{n\left(N-1\right)}\right] \end{array} \end{array}$$

where *N* is the population size, *r* is the fraction of responses that the author was interested in, *x* is the number of ‘positive' observations (e.g., the number of people out of the *n* sampled people based on the inclusion criteria), and *Z*(*c*/100) is the critical value for the confidence level *c*.

Additional sixteen [12] female basic school pupils who admitted to having ever been pregnant whilst in school were also purposively selected for an in-depth interview using a structured interview guide to further explore the research topic. This makes a total respondent of four hundred and twenty-seven (427) basic school pupils in the study.

### 3.2. Data Collection and Data Analysis

In order to retain the appropriate language, sequencing of questions, and cultural flavor, the research instruments were pretested in another municipality (Agona West Municipal), which has similar characteristics as that of the Effutu Municipality. The lessons learned from the pretest were used to finalize the research instruments. Data collection commenced after obtaining ethical clearance and informed consent from the selected participants and written approval from all the 49 basic schools in the study area. The data was collected via one-on-one interviews using five [5] field assistants purposely trained for the study. The content of the field assistant training focused on the study objectives, familiarity with the research instruments, approaches to credible data collection, data transcription, and ethical issues relating to young people's sexual and reproductive health decisions and research integrity. Due to the voluntary nature of participation in the study, participants were not evenly distributed across the schools most especially when in some schools the level of participation was less than 5 pupils.

Quantitative data were analyzed using the Statistical Package for Social Sciences (SPSS) and results presented using descriptive statistics to identify differences in contraceptive practices and challenges among the different age groups of respondents. Content analysis was used in analyzing the in-depth interview responses. Data analysis began with data cleaning through the identification and removal of inaccurate data from the data set to maintain consistent and accurate data. Once the quantitative data are collected, responses to each question were assigned a numeric value so that the information can be uploaded and analyzed using the SPSS software. For the qualitative data, the interviews were audiotaped with permission from participants to ascertain accurate accounts of the interviews. The recordings were then replayed for analytical responses. Interviews were transcribed immediately thereafter. All interviews were conducted in English.

## 4. Results

In Table 1, the author presents the background characteristics of the respondents. The majority of respondents (56%) were aged 13–15years, in JHS (72%), living with both parents (50%) and were the first child of their parents (56%).

The sexual relationships of respondents were presented in Table 2. It was observed that about 67% of the respondents reported to having been in a relationship and had ever had sexual intercourse in the relationship (60%). Majority (86%) of their sexual partners were aged 16–18 years and are pupils (99%), and their first sexual encounters in the relationship were reported as on own will (80%).

Some respondents narrated various reasons and experiences with their relationships during the in-depth interviews. Predominantly, the reasons for indulging in boy-girl relationships were for material support and companionship. One of such respondents indicated that, *“I have two boyfriends, one is my senior in school and the other guy is a mechanic in town. My senior helps me with school work whilst the other guy gives me money and sometimes buys me things when my parents don't have. I sometimes have sex with them when they beg me for it”* (15 years, JHS 2 girl).

Another respondent indicated that, *“we are very close friends in the same class and loved each other. ‘Sometimes we study together, visit each other at home and go out to have fun. We only had sex once in his house whilst watching a movie during vacation but it was a mistake and we promised each other it will not happen again”* (13 years, primary 6 girl).

Another respondent said, *“I have been with my guy since primary 5 and he is a gentle guy. We are both in the same areal and share things together. Sometimes, he gives me money when I don't have and advise me. We have sex anytime we are together in a close place and start touching each other”* (14 years, JHS 1 girl).

The author presents respondents' awareness and use of contraceptives in Table 3. Respondents' awareness and use of contraceptives varied with about 65% reported to have ever heard of a contraceptive method and 21% ever used a modern contraceptive predominantly an emergency contraceptive pill (48%). Television (TV) was the main reported source of information on contraceptives (33%) and respondents' parents, relatives, and guardians were the main sources of contraceptives used (72%). Regarding condom use during most recent sexual intercourse, about 83% of respondents reported having tried using a condom. The majority (71%) however indicated that they started with condoms but ended ‘raw.' For respondents who used condoms, about 86% indicated it was a joint decision to do so.

Discussions during the in-depth interview show a discrepancy between awareness and use of contraceptives among the respondents. Whereas there is a high level of contraceptive awareness among respondents, usage was discretional. Some respondents explained that *“know about many contraceptives but I learned when you don't have a child and start using contraceptives it will make you infertile in future so for me and my guy we only use condoms, but sometimes he complains it's not nice so he removes the condom and says he will release outside so that I don't get pregnant”* (15 years, JHS 3 girl).

For those who have ever used emergency contraceptives, they believe it is the best contraceptive method since its use only when one has sex. A respondent indicated that, *“we saw the emergency contraceptive advert on TV and think it's good so my man always gives me Lydia or Postinor11 to take as soon as we have sex. Because I'm afraid of unwanted pregnancy, I take it soon after every round of sex”* (16 years, JHS 3 girl). Some respondents said they do not have sex without a condom no matter who or what it is. Two of such respondents recall a local Ghanaian TV advert on condom use and emphatically indicated that, *“no condom no sex”* (15 years, JHS 3 girl) and *“if a condom is not on, I won allow him to entre me”* (14 years, JHS 2 girl).

**Figure 1:**
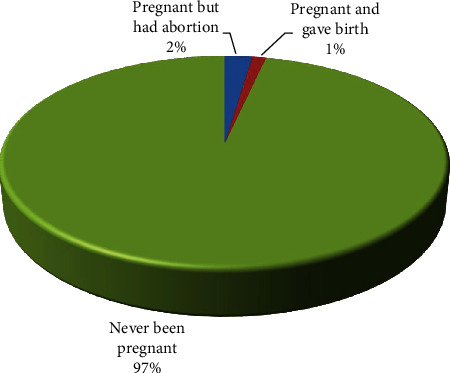
History of unplanned pregnancy and induced abortion. Source: field data 2019.

There were also reported histories of unwanted pregnancies (4%) resulting in unplanned childbirths (1%) and induced abortions (2%) by respondents in some instances (Figure 1).

For these respondents, they first heard about contraceptives and started using them following an unwanted pregnancy leading to childbirth or after an induced abortion. Four [4] respondents in JHS claimed they had ones been pregnant and had an abortion after which the service provider in consultation with their parents introduced them to contraceptives to prevent future unwanted pregnancies.

One of these respondents indicated that, *“I became pregnant in JHS 1 but didn't know it was pregnancy until my teacher noticed it and informed my mom who took me to a clinic for an abortion after which they forced me to take an injection every 3months so that I don't become pregnant again”* (15 years, JHS 2 girl). Another claimed, “*my stepmom forced me to have an abortion when she noticed I was pregnant and was later given a family planning drug to prevent me from becoming pregnant again so that I can complete school”* (15 years, JHS 5 girl). Another respondent said, *“When my auntie suspected I was pregnant, she took me to a hospital for abortion and some family planning thing under my arm so that I will not get pregnant again. The nurse said it is called implant which will protect me for five years”* (14 years, JHS 1 girl).

## 5. Discussion

The Ghana government developed a national population policy in 1960, an adolescent reproductive health policy in 2000, and a national HIV/AIDS and STI policy in 2004 among others to respond to the sexual and reproductive health needs of adolescents and young people [13]. Despite the existence of these policies and other programs, Ghana continues to record high incidences of teenage pregnancies resulting in unwanted teenage births and unsafe abortions which can be attributed to lack of sexual abstinence and/or low prevalence of modern contraceptive use among others [14]. Some nationwide studies [15–17] that investigated contraceptive use among young people in Ghana have been too general, used quantitative methods, and/or focused on prevalence thereby limiting empirical evidence which is a gap that the current study attempted to fill by investigating contraceptive use among basic school pupils in the study area to further inform policy and program decisions in Ghana.

The current research was a case study of contraceptive use among basic school pupils of a municipality in Ghana. The researchers focused on respondents' background characteristics, sexual relationships, pregnancy/induced abortion history, and awareness and use of contraceptives. From the study results, the majority of respondents (56%) were aged 13–15years, in JHS (72%), were living with both parents (50%), and were the first child of their parents (56%). About 67% reported being in a relationship and had ever had sexual intercourse in the relationship (60%). Majority (86%) of their sexual partners were aged 16–18years and were pupils (99%). Moreover, their first sexual encounters in the relationship were reported as on their own will (80%).

Respondents' awareness and use of contraceptives varied with about 65% reported to have ever heard of a contraceptive method and 21% ever used a modern contraceptive predominantly an emergency contraceptive pill (48%). Television (TV) was the main reported source of information on contraceptives (33%), and respondents' parents, relatives, and guardians were the main sources of contraceptives used (72%). Regarding condom use during most recent sexual intercourse, about 83% of respondents reported having tried using a condom. The majority (71%) however indicated that they started with condoms but ended ‘raw.' For respondents who used condoms, about 86% indicated it was a joint decision to do so.

Contrary to public perceptions that basic school pupils in Ghana are too young to indulge in sexual relationships [12], the study results show that basic school pupils are in sexual relationships and have various reasons and experiences other than financial/material gains. The beginning of such relationships although started with platonic friendship, intimacy set in when the opportunity came to be alone in enclosed environments without parental/adult presence.

Although there is universal awareness of contraceptives in Ghana [18], little information exists on the situation among basic school pupils. The existing discrepancy between awareness and use of contraceptives among the respondents in this study shows that contraceptive information education and communication are not targeted at basic school pupils. In situations where awareness exists, the source of information is predominantly from peers whose credibility and authenticity of the correctness of the information received cannot be guaranteed thereby making contraceptive use among these age groups more discretional with the associated consequences.

The observation that some basic school pupil first heard about contraceptives and started using them following an unwanted pregnancy, childbirth, or after an induced abortion confirms the relevance of the proposed comprehensive sexuality education by the Nana Ado Dankwa Affu Addo's lead government in 2019 which aimed at educating school children at the basic school levels of sexuality and health choices. Unfortunately, this proposal was aggressively received with religious and sociocultural sentiments that aborted the implementation.

Respondents in the JHS were more sexually active than those in the primary school level. However, those sexually active pupils who received education on contraceptive use from their parents/guardians are more likely to use contraceptives consistently compared to their counterparts who do not. Consequently, awareness and use of contraceptives were more prevalent among the JHS pupils compared to those in the upper primary. There were reported instances of unwanted pregnancies that resulted in abortion which marked the beginning of contraceptive use. Teenage girls who get pregnant are likely to drop out of school and are unlikely to have the social and economic means to raise children [19]. Further, unintended pregnancy poses a major challenge to the reproductive health of young adults in developing countries. The researcher observed that sexual activity among the respondents is most of the time unprotected leading to unwanted pregnancies and invariably abortion. This situation has also been reported as common in countries where there is a persistently high rate of unmet need for family planning and low rates of contraceptive use among young people, particularly in underdeveloped or developing countries [20].

According to the World Health Organization, 2011, approximately 14-15 million teenage girls and young women become mothers every year and this accounts for more than 10% of births worldwide. Teenage pregnancy is a serious public health issue worldwide. Sexual activities among adolescents in sub-Saharan Africa occur mostly at the age of 15 years; this sexual debut develops at a time when the adolescent has insufficient knowledge about contraceptives and their use which expose them to high risks of STIs and unwanted pregnancies. Following the efforts to achieve the millennium development goals (MDGs) 5 and the succeeding sustainable development goal (SDG) 3, Adolescent sexual and reproductive health (ASRH) issues have gained more prominence and become a global concern. [10] This emphasizes the crucial role of adolescent reproductive health research in achieving sustainable development goal 3.

In the case of developed countries compared to developing and underdeveloped countries, teenage pregnancy rates have been reduced because of the intense campaign, education, and subsequent uptake of contraceptives by young people without any form of social hindrances [21]. For instance, between 1990 and 2008, pregnancy among teenagers in the United States of America reduced from 117 pregnancies per 1,000 women aged 15–19 to 67 per 1,000 women, representing a 42 percent drop in teenage pregnancy [22, 23]. Analysis of data from the National Survey of Family Growth (NSFG), a major source of government data on population and reproductive health in the US found that 86 percent of the decline in teen pregnancy rates in 2002 occurred due to improved uptake of contraceptives among adolescents. Evidence-based interventions in family planning suggest that referring young adolescents to family planning clinic for contraceptives significantly improve contraceptive acceptance and use among this group [24].

In Ghana, adolescent sexual and reproductive health has over the years been a great concern to stakeholders in the field due to the early commencement of sexual activity, poor knowledge of contraceptives, and limited access to and underutilization of reproductive health services [25]. Poor knowledge of contraceptives among adolescents could result in exposure to inaccurate or incomplete information which may lead to the prevalence of sexually transmitted infections (STIs), unwanted pregnancies, and related health problems [26].

Evidence exists that sexual activities among young adolescents in Ghana are most times unprotected leading to unwanted pregnancies and invariably complications of unsafe abortions [27, 28]. The high prevalence of unwanted pregnancy among teenagers in Ghana and the upsurge of HIV infection in the country pose a serious challenge to the Ghana Health Service, the Ghana Education Service, and relevant stakeholders. This situation underscores the critical need to explore awareness, knowledge, and contraceptive use among young adolescents in basic schools to inform policy and program interventions.

Evidence from Ghana also shows that older adolescents who receive education on contraceptive use from their parents are more likely to use contraceptives consistently compared to their counterparts who do not [29]. Despite the many setbacks to the implementation and delivery of comprehensive sexuality education (CSE) in basic schools in Ghana, the urgency of finalizing and implementing this policy in basic schools in Ghana is greater than ever before.

## 6. Conclusion

In this study, the researcher explored contraceptive use among basic school pupils in a Ghanaian municipality. The results show that basic school pupils in Ghana are generally sexually active but have an unmet need for modern contraception due to sociocultural reasons, stigma, and misconceptions about contraceptives. Contraceptive awareness and use are more prevalent among junior high school pupils compared to those at the primary levels. Pupils who received contraceptive education from parents/guardians were, however, more likely to use modern contraceptives consistently compared to their counterparts who do not. In light of recent controversies surrounding comprehensive sexuality education in Ghana, the researcher's belief findings of this study provide some empirical evidence to justify national policy and program decisions on introducing comprehensive sexuality education in Schools.

The researcher, therefore, recommend that contraceptive education should be introduced at the basic school level in Ghana, where young people can be educated by trained professionals and guided to make informed reproductive health choices in life. In this regard, there is a need for multisectoral collaboration and more commitment to increased local funding by the government and development partners in Ghana on contraceptive issues relating to young people to ensure the desired results.

## Figures and Tables

**Table 1:** Background characteristics of respondents.

Variable	Frequency	Percent (%)
Age		
10–12	65	15.2
13–15	237	55.5
16–18	125	29.3
Total	427	100
Class		
Upper primary	118	27.6
JHS	309	72.4
Total	427	100
Religion		
Christian	372	87.1
Islam	46	10.8
Traditionalist	9	2.1
Total	427	100
Sex		
Female	224	52.5
Male	203	47.5
Total	427	100
Ethnicity		
Fante	286	67.0
Akan	30	7.0
Ewe	51	12.0
Ga-Adangbe	34	8.0
Others	26	6.1
Total	427	100
Person leaving with		
Live alone	20	4.7
Both parents	212	49.7
Mother only	140	32.8
Father only	32	7.5
Others	23	5.4
Total	427	100
Number of siblings		
0	174	40.8
1	17	4.0
2	115	27
3	119	27.9
>4	2	0.5
Total	427	100
Birth order among siblings		
1	238	55.7
2	50	11.7
3	58	13.6
4	50	11.7
>5	31	7.3
Total	427	100

Source: field data 2019.

**Table 2:** Sexual relationships.

Variable	Frequency	Percent (%)
Do you have a boyfriend/girlfriend?		
Yes	285	66.8
No	115	26.9
Neutral	27	6.3
Total	427	100
Age of boyfriend/girlfriend (for yes responses)		
10–12	57	13.4
13–15	2	0.5
16–18	366	85.7
>18	2	0.5
Total	427	100
Occupation of boyfriend/girlfriend		
Student	424	99.3
Teacher	2	0.5
Mechanic	1	0.2
Total	427	100
Ever had sex with boyfriend/girlfriend		
Yes	256	60.0
No	169	39.6
Neutral	2	0.5
Total	427	100
First sex description		
Own will	339	79.4
Coaxed	43	10.1
Forced	32	7.5
Neutral	13	3.1
Total	427	100

Source: field data 2019.

**Table 3:** Respondents' awareness and use of contraceptives.

Variable	Frequency	Percent (%)
Ever heard about contraceptives?		
Yes	276	64.6
No	115	27
Neutral	36	8.4
Total	427	100
Source of information (for yes responses)		
TV	141	33.0
Radio	58	13.6
Parents	17	4.0
Peers	27	6.3
Health workers	47	11.0
Others	137	32.1
Total	427	100
Ever used modern contraceptives		
Yes	91	21.3
No	334	78.2
Neutral	2	0.5
Total	427	100
Which contraceptives ever used		
Condoms	63	14.8
Emergency contraceptive pills	203	47.5
Injectable	43	10.1
Implants	19	4.5
Oral pills	31	7.3
Others	56	13.1
Neutral	12	2.8
Total	427	100
Source of contraceptive ever used		
Pharmacy/drug store	60	14.1
Friends	35	8.2
Sexual partner	10	2.3
Hospital/family planning clinic	14	3.3
Others (parents, relatives, or guardians)	308	72.1
Total	427	100
Condom use during most resent sexual intercourse		
Yes	53	12.4
No	71	16.6
Others (started with condom but ended raw)	303	71.0
Total	427	100
Who suggested use of condom		
Myself	44	10.3
Partner	18	4.2
Joint decision	365	85.5
Total	427	100

Source: field data 2019.

## Data Availability

Data for the study can be obtained from the author upon reasonable request.

## Abbreviations

AIDS: Acquired immunodeficiency syndrome
ASRH: Adolescent sexual and reproductive health
GDHS: Ghana Demographic and Health Survey
HIV: Human immunodeficiency virus
IRB: Institutional Review Board
JHS: Junior high school
MDGs: Millennium development goals
NPC: National Population Council
NSFG: National Survey of Family Growth
RH: Reproductive health
SDG: Sustainable development goals
SPSS: Statistical Package for the Social Sciences
STIs: Sexually transmitted infections
TV: Television
US: United States
WHO: World Health Organization.
